# Editorial: Natural products as an emerging therapeutic alternative for the treatment of anxiety and depression, Volume II

**DOI:** 10.3389/fphar.2022.1078548

**Published:** 2022-12-14

**Authors:** Ajmal Khan, Haroon Khan, Francesco Maione, Nasiara Karim

**Affiliations:** ^1^ Natural and Medical Sciences Research Center, University of Nizwa, Nizwa, Oman; ^2^ Department of Pharmacy, Abdul Wali Khan University, Mardan, Pakistan; ^3^ ImmunoPharmaLab, Department of Pharmacy, School of Medicine and Surgery, University of Naples Federico II, Naples, Italy; ^4^ Department of Pharmacy, University of Peshawar, Peshawar, Pakistan

**Keywords:** natural products, depression, major depressive disorder (DEP), monoamine oxidase (MAO), herbomics

Currently anxiety and depression are the fourth leading cause of morbidity. Anxiety is the most common mental disorder from which 20% of the adult population suffer worldwide and has become a significant research area in the field of psychopharmacology (Yadav et al., 2008; Sharmen et al., 2014). Anxiety is also associated with significant disability resulting in negative impacts on the patient’s quality of life (Kudagi et al., 2012). Currently, Benzodiazepines are used as the drug of choice for the treatment of several types of anxiety disorders (Gupta et al., 2010). Despite the fact that BZPs have known advantages but their side effects are high including sedation, myorelexation, physical dependence and anterograde amnesia (Barua et al., 2009) which has limited their use.

Major depressive disorder (MDD) is also one of the most common psychiatric disorders, posing serious public health problem (Kessler et al., 2005). According to WHO, the prevalence of depression is approximately 4.5% globally with almost 325 million people suffering from it (WHO, 2017). The classical antidepressant drugs available on the market for treating depression include tricyclic antidepressants, monoamine oxidase (MAO) inhibitors, and selective serotonin reuptake inhibitor drugs. Although these drugs are effective in treating patients with depression, however there are still almost 50% of patients who are resistant to the first line conventional antidepressant drugs. Additionally, these drugs require almost 3–4 weeks before showing therapeutic outcome (Browne and Lucki, 2013). Furthermore, the classical antidepressant drugs are associated with serious side effects such as sedation, dry mouth, restlessness, muscle spasms, nausea, constipation, profuse sweating, sexual disorders, obesity, confusion, and increased suicidal thoughts over a prolong period of time (Schosser et al., 2012; Bet et al., 2013). This burden is especially high in many low and middle income countries.

In the last few decades have seen a significant rise in the use of natural remedies to treat various ailments including depression and anxiety. These products are perceived as safer alternatives to pharmacotherapy, with lower risk of adverse effects or withdrawal. Considerable efforts have been made in recent years to discover substances from natural sources particularly plants which can help prevent these serious mental disorders. Natural products are small molecules present in divergent natural sources. They are considered to possess one of the most coveted positions in the treatment of all human disorders including anxiety and depression. They are considered to be the most important source of novel drug leads (Nasri et al., 2014). The importance of plant derived natural products for the treatment of anxiety and depression is evident from the fact that many herbal remedies have been reported (Sarris et al., 2013; Farah et al., 2016). Thus, due to an increasing interest, herbal medicines coupled with the use of emerging genetic technologies “herbomics,” are potential areas of future research.

In view of the above, there is a dire need for the discovery and development of novel antidepressant agents acting *via* different mechanisms that may provide quick onset of action for relieving anxiety and depression symptomatology. Furthermore the newer agents may prevent the aforementioned adverse effects and provide effective treatment to the non-responsive patients to the conventional drugs.

This Research Topic focuses on original contributions for natural products being useful in various mental disorders particularly anxiety and their possible mechanisms of action.
